# Physical activity level and health-related quality of life in adults with multiple osteochondromas: a Dutch cross-sectional study

**DOI:** 10.1038/s41598-025-02812-3

**Published:** 2025-05-30

**Authors:** Ihsane Amajjar, Kuni Vergauwen, Nienke W. Willigenburg, Ivan P. J. Huijnen, Rob J. E. M. Smeets, S. John Ham

**Affiliations:** 1 Department of Orthopedic Surgery, OLVG, Oosterpark 9, 1091 AC Amsterdam, The Netherlands; 2 Department of Rehabilitation Medicine, CAPHRI, Maastricht University, Maastricht, The Netherlands; 3 Department of Health Care, AP University College, Antwerp, Belgium; 4 MOVANT, Department of Rehabilitation Sciences and Physiotherapy, University of Antwerp, Antwerp, Belgium; 5 Adelante, Centre of Expertise in Rehabilitation and Audiology, Hoensbroek, The Netherlands; 6 Zuyd University of Applied Sciences, Heerlen, The Netherlands; 7 Pain in Motion International Research Group, Brussels, Belgium; 8 Clinics in Rehabilitation, Eindhoven, The Netherlands

**Keywords:** Quality of life, Exercise, Multiple Hereditary Exostoses, Multiple osteochondromas, Physical activity, Health care, Quality of life, Bone cancer

## Abstract

Multiple Osteochondromas (MO) can significantly impact physical functioning, yet evidence on how MO affects physical activity levels (PAL) and health-related quality of life (HRQOL) remains limited. This study aimed to: (1) characterize the PAL and physical and mental HRQOL of adult patients with MO and compare them with healthy subjects (2) explore whether illness-related symptoms, sociodemographic and psychological factors are associated with patients’ PAL and HRQOL. This cross-sectional study used a survey consisting of sociodemographic data and validated questionnaires on the PAL (Baecke Physical Activity Questionnaire) and HRQOL (SF-36). The PAL, physical and mental HRQOL were compared with reference scores of healthy subjects using a one-sample t-test. An a-priori defined theoretical framework (ICF-model) was used to select explanatory variables, including several psychological factors, for the multiple linear regression models of the dependent variables PAL and HRQOL. 342 patients (42.6% males) with a mean age of 41.8 ± 16.3 completed the survey. Mean PAL scores were 7.2 ± 1.7, physical HRQOL 41.7 ± 11.1 and mental HRQOL 49.1 ± 10.5. Except for mental HRQOL, these scores were lower than healthy subjects (*p* < 0.001). The final regression model for the PAL contained six factors (R^2^ = 0.221, *p* < 0.001) showing the strongest association with having a job and malignant degeneration. Fourteen variables, including pain-related disability and number of surgical procedures, explained physical HRQOL (R^2^ = 0.731, *p* < 0.001). For mental HRQOL, eight factors remained in the model (R^2^ = 0.618, *p* < 0.001) with anxiety explaining the most unique variance (9.4%). MO patients reported significantly lower PAL and physical HRQOL than healthy controls. This study provides insight in several factors associated with the PAL and HRQOL in MO which could be used to optimize patients’ treatment.

**Supplementary Information:**

The online version contains supplementary material available at 10.1038/s41598-025-02812-3.

## Introduction

Multiple Osteochondromas (MO), or Hereditary Multiple Exostoses (HME), is a rare autosomal dominant inherited skeletal disorder characterized by multiple benign bone tumors, typically developing at the metaphysis of long bones and axial skeleton^1,2^. Its prevalence in Western countries has been estimated at 1:50,000. in previous literature^2,3^, with an equal distribution between genders^4^. Osteochondromas in MO can cause various complications such as compression on surrounding blood vessels, peripheral nerves and muscles, interference with growth, reduced mobility, and pain. Malignant degeneration of osteochondromas has been reported in 9.3% of patients aged 16 and older^5^. Approximately 66–88% of the patients will undergo at least one surgical procedure because of this disorder^2,3,6^.

Literature to date has mainly focused on the origin of MO, surgical procedures or specification of deformities^1,3,7^. Only a few studies focused on health-related quality of life (HRQOL) issues, activity limitations, or perceived symptoms associated with MO^3,8,9^. Patients with MO have reported lower HRQOL compared to the general population^3,8^. In other chronic conditions, various sociodemographic factors (comorbidities^10^, gender^10–12^, age, educational level^10–13^, BMI^11,12^, relationship status^14^, illness-related (pain and fatigue intensity^11–13,15–18^) and psychological factors (fear-avoidance beliefs, pain catastrophizing, depression, anxiety)^11,13,15,17,18^ are associated with patients’ perceived physical disability, physical activity level (PAL) and HRQOL. To our knowledge, only prevalence of pain and fatigue was investigated in patients with MO, with pain being reported by 76–95%^6,16,19^ and severe fatigue by 71%^19^.

Preceding literature on gender differences in HRQOL of MO-patients has presented contradicting findings. The study of D’Ambrosi et al.^9^ found that particularly female patients have a lower physical HRQOL, whereas other studies could not find differences between females and males^7,14^. More research is necessary to determine the role of gender in MO. No studies were identified that specifically explored sociodemographic, illness-related or psychological determinants of HRQOL in patients with MO. The lack of insight into psychosocial symptoms and their potential impact on HRQOL underscores the need for additional studies in this population.

Activity limitations, defined by International Classification of Functioning, disability and health (ICF) as difficulties in executing activities^20^, have been reported as a consequence of chronic pain; 26–56% of the MO-patients experienced problems during occupation^3,8^ and 7% was unable to work^6^. Patients also reported occupational problems due to pain with a great impact on QOL and negative effect of other MO-related problems on participation in sports^3^. However, no studies to date evaluated PAL in MO, defined as any bodily movement produced by skeletal muscles resulting in energy expenditure^21^. Physical activity is an important lifestyle factor in the prevention of major non-communicable disease and premature mortality^22^, but also reduces psychological burden and enhances overall well-being^23^.

In healthy controls and various other chronic conditions, a higher PAL was found to be associated with a higher HRQOL^11,24,25^. The directionality and strength of these associations can differ depending on the patient population, emphasizing the need to explore these factors in each specific chronic condition.

Given these knowledge gaps in MO, the present study aims to characterize patients’ PAL, HRQOL and to explore whether illness-related symptoms, sociodemographic or psychological factors are associated with patients’ PAL and HRQOL.

Based on previous research findings five hypotheses were formulated:

1. patients with MO have a lower HRQOL and PAL than healthy controls.
2. a higher PAL, after controlling for other factors, is significantly associated with a higher HRQOL in patients with MO.
3. higher BMI, higher pain and fatigue, and presence of psychological factors are negatively associated with the PAL.
4. female gender, higher BMI, comorbidity, higher pain and fatigue, physical disability and presence of psychological factors are negatively associated with physical HRQOL.
5. female gender, being single, a lower educational level, malignancy, more surgical interventions, higher intensity level of pain and fatigue, and presence of psychological factors are negatively associated with mental HRQOL.

## Methods

### Study design and patients

This cross-sectional study was performed in the Netherlands between May 2018 and December 2019 using an online survey. Adult Dutch-speaking patients diagnosed with MO, who were treated at the orthopedic outpatient clinic of the national HME-MO-expertise center (OLVG, Amsterdam) and members of the Dutch Patient Association ‘HME-MO Vereniging Nederland’, were invited to participate. Patients were recruited during their outpatient clinic visit or contacted by telephone. Members of the Dutch Patient Association could contact the coordinating researcher (IA) for more information by telephone or email via contact information provided through an online post on the Patient Association’s website. If interested, a patient information letter and informed consent form were sent by post or e-mail. This study was reported according to the Checklist for Reporting Results of Internet E-Surveys (CHERRIES) guidelines, see Appendix 2^26^.

### Data collection

After providing informed consent, patients received a secure link by e-mail granting access to a digital questionnaire in Castor, Electronic Data Capture^27^. If patients had no access to the internet or a computer, a paper survey was sent by post. The survey consisted of a sociodemographic section and validated Dutch questionnaires regarding physical activity, quality of life, pain, fatigue and psychosocial factors.

#### Primary outcomes (dependent variables)

The physical activity level was measured with the Baecke Physical Activity Questionnaire (BPAQ), which was validated in a Dutch population^28^. The total score ranges from 3 to 15 and a higher score indicates a higher level of physical activity.

Health-related quality of life was measured with the Medical Outcomes Study Short-Form 36 (SF-36)^29,30^. The physical component (SF-36 PCS) and mental component scores (SF-36 MCS) were calculated according to their specific instructions^29,30^. Subscale scores range from 0 to 100 and a higher score indicates higher levels of well-being and lower bodily pain^29,31^.

The BPAQ and SF-36 scores were compared to reference scores of the Dutch general population^28,29^.

#### Explanatory (independent) variables

Sociodemographic information as listed in Table 1 was collected. Pain was assessed using an 11-point numeric rating scale (NRS) for the patient’s average pain severity during the previous week. A higher score indicates greater pain severity, ranging from 0 to 10^32^. The Pain Disability Index (PDI) was used to measure for the interference of average pain complaints on functioning^33^. Scores range from 0 to 70, with higher values indicating greater interference. The Douleur Neuropathique en 4 Questions (DN4) was used for neuropathic pain. The DN4 consists of two parts, a questionnaire assessing pain characteristics and symptoms of abnormal sensations, and a clinical examination^34^. In this study, only the questionnaire was used, where a score of 4 or above (range 0–7) indicates neuropathic pain^34^.

**Table 1:** Sample description.

Characteristics	Total (n = 342)	Men (n = 146)	Women (n = 196)	*p*-values
Marital status (%)				
Single	14.9	15.1	14.8	0.944
In a relationship	79	81.5	77	0.676
Divorced	3.8	2.1	5.1	0.122
Widowed	2.3	1.4	3.1	0.281
Educational level (%)				
Primary	0.6	0.7	0.5	0.833
Secondary	56.4	56.2	56.6	0.931
Tertiary	40.6	41.1	40.3	0.883
Other	2.3	2.1	2.6	0.765
Paid work (%)	66.7	69.9	64.3	0.278
Surgery (%)				
0–2	30.4	34.2	27.6	0.183
3–5	24.3	19.9	27.6	0.096
6–10	22.5	21.9	23	0.820
> 10	22.8	24	21.9	0.659
Comorbidity (%)	24.9	18.5	29.6	0.023
Family members with MO (%)	81.6	80.8	82.1	0.756
Malignancy of MO (%)	8.8	11	7.1	0.232
Pain locations (%)				
No pain	11.7	17.8	7.1	< 0.002
1–2	23.7	31.5	17.9	< 0.005
3–4	21.6	19.9	23.0	0.493
5–7	22.8	22.6	23.0	0.938
> 7	20.2	8.2	29.1	< 0.001
Positive DN4 (%)	29.2	23.3	33.7	0.041

	Mean (SD)	Mean (SD)	Mean (SD)	
Age (y)	41.8 ± 16.3 (17–91)	41.4 ± 16.5 (17–85)	42 ± 16.3 (18–91)	0.756
BMI	26 ± 5.2 (16.2–44.5)^a^	26 ± 4.7 (17.7–44.1)^a^	26.1 ± 5.5 (16.2–44.5)	0.851
Disease duration (years)	31.7 ± 17.1 (0–81)^a^	30.4 ± 18.1 (0–81)	32.6 ± 16.3 (1–81)^a^	0.232
Fatigue (NRS)	4.1 ± 2.6 (0–10)	3.4 ± 2.5 (0–10)	4.7 ± 2.5 (0–10)	< 0.001
CIS	84.1 ± 15.4 (35–123)	82.1 ± 16.2 (35–123)	85.7 ± 14.6 (49–122)	< 0.036
Pain (NRS)	3.2 ± 2.6 (0–10)	2.6 ± 2.4 (0–8)	3.7 ± 2.6 (0–10)	< 0.001
PDI	17.2 ± 14.9 (0–65)	13.7 ± 14 (0–58)	19.8 ± 15.1 (0–65)	< 0.001
PCS	12.8 ± 9.4 (0–46)	11.9 ± 9.4 (0–46)	13.6 ± 9.4 (0–42)	0.095
BPAQ total score	7.2 ± 1.7(0.8–10.6)	7.2 ± 1.8 (0.8–10.5)	7.1 ± 1.7 (0.9–10.6)	0.762
Work	2.4 ± 0.8 (0.8–4.8)	2.3 ± 0.9 (0.8–4.8)	2.4 ± 0.7 (0.8–4)	0.565
Sport	2.2 ± 0.9 (0–4.3)	2.2 ± 0.9 (0–4.3)	2.1 ± 0.9 (0–4)	0.137
Leisure time	2.6 ± 0.9 (0–4.5)	2.6 ± 0.9 (0–4.5)	2.7 ± 0.9 (0–4.5)	0.723
SF-36 physical component score	41.7 ± 11.1 (10.8–62.9)	44.6 ± 10.7 (16.3–61.6)	39.6 ± 10.9 (10.8–62.9)	< 0.001
SF-36 mental component score	49.1 ± 10.5 (14.4–66.5)	50.4 ± 10.0 (14.4–64.5)	48.2 ± 10.8 (14.8–66.5)	0.064
HADS anxiety	5.7 ± 3.9 (0–20)	4.9 ± 3.7 (0–18)	6.4 ± 4.0 (0–20)	< 0.001
HADS depression	3.8 ± 3.5 (0–20)	3.8 ± 3.6 (0–20)	3.8 ± 3.4(0–18)	0.911
FABQ	31.5 ± 20.6 (0–92)	29.4 ± 21.8 (0–92)	33.1 ± 19.6 (0–84)	0.102

Values are mean ± SD (range) unless noted otherwise.

^a^data missing from 1 participant.

*DN4* douleur neuropathique en 4 questions, *NRS* numeric rating scale, *CIS* checklist individual strength, *PDI* pain disability index, *PCS* pain catastrophizing scale, *SF-36,*short form-36, *BPAQ* Baecke Physical Activity Questionnaire, *HADS* Hospital Anxiety and Depression Scale, *FABQ* Fear-Avoidance Beliefs Questionnaire.

Fatigue measures included an 11-point NRS for the patient’s average fatigue severity of the previous week, with a higher score indicating higher severity^35^. The Checklist Individual Strength (CIS) measures four areas of fatigue: fatigue severity, concentration, motivation and activity^36^. In this study, the total score of the CIS was used, ranging from 20 to 140, with higher scores indicating greater fatigue^36^.

Included psychological factors were anxiety and depression complaints measured with the Hospital Anxiety and Depression Scale (HADS) where higher scores indicate more severe symptoms (range 0–21)^37,38^. The Pain Catastrophizing Scale (PCS) measures patients’ catastrophizing thoughts and feelings related to pain^39^. The total score ranges from 0 to 52, with higher values indicating greater catastrophizing^40^. Fear-avoidance beliefs regarding physical and work-related activities were measured with the Fear Avoidance Beliefs Questionnaire (FABQ). Scores range from 0 to 96, with higher scores reflecting stronger avoidance beliefs^41^.

Further details on the assessments are available in Appendix 1 (supplemental material).

### Statistical analysis

All data were analysed in SPSS version 27 for Windows (SPSS Inc. Headquarters, 233 s. Wacker Drive, 11th floor, Chicago, Illinois 60606, USA). The online survey had minimal risk of missing data because the digital software prevented patients from skipping a question. Patients who received a paper survey were contacted by telephone in case of missing items and the missing data were handled according to the questionnaire’s specific instructions, if available^30^. Due to insufficient available data, an a priori power calculation was not performed. The aim was to include a convenience sample of at least 300 adults, based on prevalence numbers of MO in the Netherlands^42^ and the findings from Goud et al. in their Dutch cohort study^3^.

Literature suggests a prevalence rate for MO of approximately 1 in 50,000 in Western populations^2^, while others have implied a higher rate^3^. Given the country’s population of 17 million, this estimate implies at least 340 affected individuals. Enrolling at least 300 adult patients and adhering to the rule of thumb of 10–15 participants per variable in linear regression modeling was intended to provide sufficient statistical power. Due to ambiguity regarding gender differences in patients with MO, descriptive data were compared between genders with a Chi-square test or independent samples t-test.

A one-sample t-test was performed to compare the normally distributed PAL (BPAQ) and HRQOL (SF-36) data of MO patients, respectively, with the reference score obtained from previous literature studies^28,29^. Patients were matched to reference scores based on gender only.

**Fig. 1:**
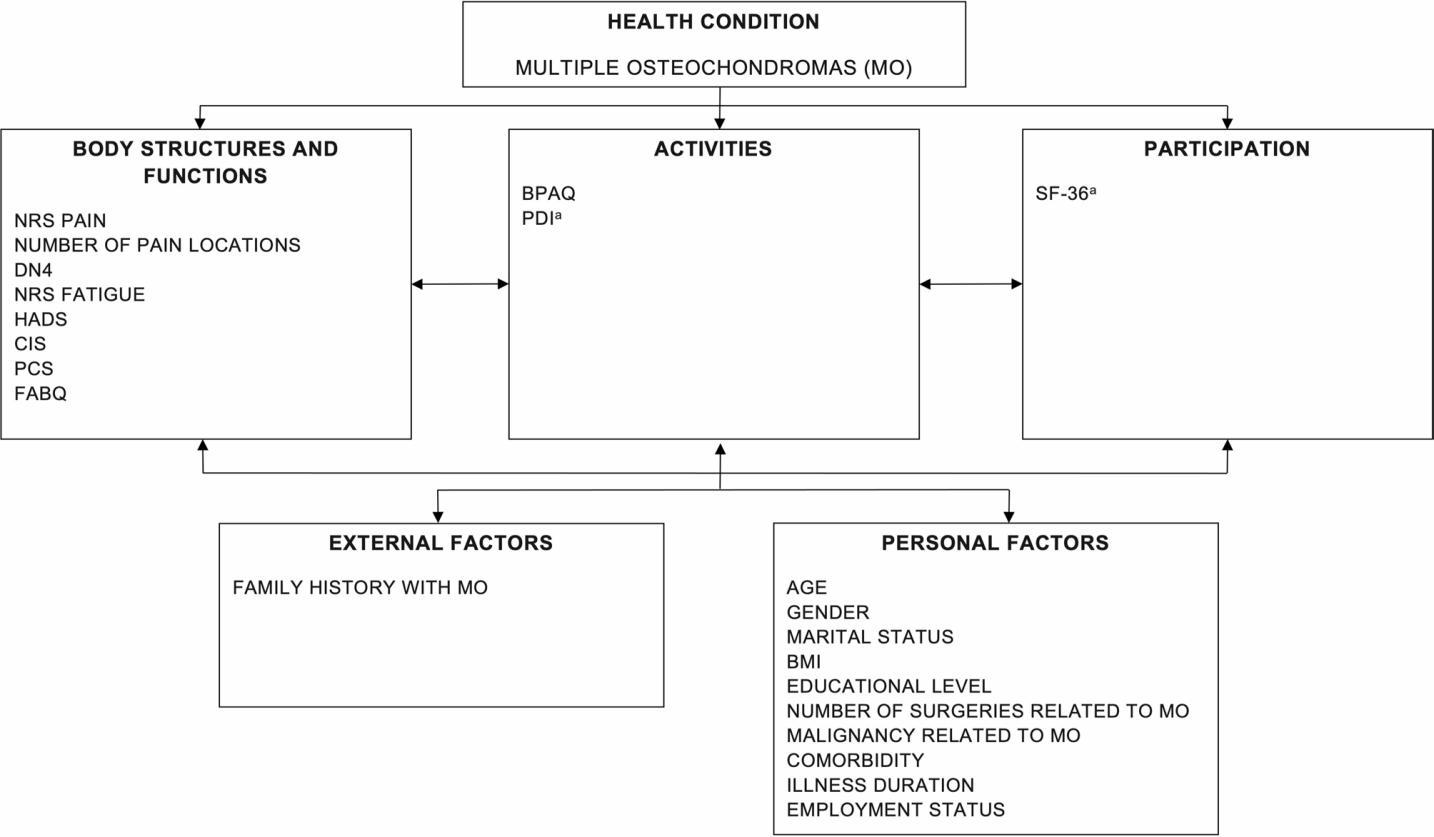
ICF model of the a priori selected explanatory variables for the dependent variables: the physical activity level (BPAQ) and physical and mental health-related quality of life (SF-36). ^a^Were not included in the regression model with the physical activity level (BPAQ) as dependent variable. The PDI measures disability at the activities level, equal to the ICF-level of the BPAQ. HRQOL, measured at the participation level, is assumed to be affected by the activities level and not the other way around. Abbreviations: *DN4* douleur neuropathique en 4 questions, *NRS* numeric rating scale, *CIS* checklist individual strength, *PDI* pain disability index, *PCS* pain catastrophizing scale, *SF-36* short form-36, *BPAQ* Baecke Physical Activity Questionnaire, *HADS* Hospital Anxiety and Depression Scale, *FABQ* Fear-Avoidance Beliefs Questionnaire, *BMI* Body Mass Index.

For the multiple linear regression models, purposeful selection of explanatory variables was performed in two steps. First, explanatory variables for all separate dependent variables (PAL, SF-36 PCS, and SF-36 MCS) were selected a priori based on a theoretical foundation i.e., the ICF model, and previous research findings on significantly associated sociodemographic, illness-related and psychological factors^11,12,14,15,17,20,42^ (Fig. 1).

Second, a priori selected explanatory variables associated with the dependent variable in univariate regression analyses at a significance level of *p* ≤ 0.25 were added to the regression model. Multicollinearity was checked for each full multiple regression model (variance inflation factor (VIF) < 10)^43^. Then, a backward multiple linear regression analysis was performed with the significance level set at *p* ≤ 0.05. Normality of residuals and homoscedasticity were checked for the final models. Additionally, we report the squared semi-partial correlation (sr^2^) for each independent variable in the final model. The sr^2^ value indicates the unique proportion of variance in the outcome explained by that specific variable after accounting for the shared variance with other variables, thereby illustrating the independent contribution of each variable to the model.

## Results

### Patient characteristics

Patients’ characteristics are presented in Table 1. A total of 342 adults with MO completed the survey. Comorbidities and neuropathic pain (DN4) were reported significantly more often by women than by men. Female patients experienced a higher pain intensity than male patients (*Mean Difference, MD* = 1.12; 95% CI [0.58, 1.66]), more pain-related disability (*MD* = 6.12; 95% CI [2.98, 9.26]), greater fatigue on both the NRS (*MD* = 1.33; 95% CI [0.8, 1.86]) and the CIS (*MD* = 3.53; 95% CI [0.24, 6.8]), a higher level of anxiety (*MD* = 1.51; 95% CI [0.68, 2.34]), and a lower physical HRQOL (*MD* = 4.98; 95% CI [2.66, 7.3]).

### Physical activity and health related quality of life in comparison to reference scores

Patients with MO reported a significantly lower mean PAL and physical HRQOL, but not mean mental HRQOL compared to reference scores of healthy, gender-matched subjects (Table 2).

**Table 2:** Comparison of means (t-test) for Baecke Physical Activity Questionnaire (PAL) and Short Form-36 (HRQOL) between reference scores of healthy, gender-matched subjects and patients with MO.

Variable		n	Reference scores (mean)	N	MO (mean ± SD)	95% CI	*p*
BPAQ work^a^	Male	139	2.6	146	2.3 (± 0.9)	2.2–2.5	< 0.001
	Female	167	2.9	196	2.4 (± 0.7)	2.3–2.5	< 0.001
BPAQ sport^a^	Male	139	2.8	146	2.2 (± 0.9)	2.1–2.4	< 0.001
	Female	167	2.4	196	2.1 (± 0.9)	2.0–2.2	< 0.001
BPAQ leisure^a^	Male	139	2.8	146	2.6 (± 0.9)	2.5–2.8	0.012
	Female	167	3.1	196	2.7 (± 0.9)	2.5–2.8	< 0.001
BPAQ total^a^	Male	139	8.2	146	7.2 (± 1.6)	6.9–7.5	< 0.001
	Female	167	8.4	196	7.1 (± 1.7)	6.9–7.4	< 0.001
SF-36 PCS^b^	Male	976	50.5	146	44.6 (± 10.7)	42.8–46.3	< 0.001
	Female	766	49.3	196	39.6 (± 10.9)	38.1–41.1	< 0.001
SF-36 MCS^b^	Male	976	51.3	146	50.4 (± 10.0)	48.7–52	0.254
	Female	766	48.4	196	48.2 (± 10.8)	46.7–49.7	0.819

*BPAQ* Baecke Physical Activity Questionnaire, *SF-36* short form-36, *PCS* physical component score, *MCS* mental component score.

^a^Reference scores from Baecke et al.^28^ (The Netherlands).

^b^Reference scores from Aaronson et al.^29^ (The Netherlands).

### Associations with the physical activity level

Table 3 presents the final regression model of PAL of patients with MO. A total of six factors remained in the model, explaining 22.1% of the total variance. The unstandardized ß shows that patients with a paid job have a 0.843 point higher PAL compared to those without a paid job. Additionally, patients who experienced more anxiety had a slightly higher PAL; for each point increase in HADS anxiety score, the BPAQ increases by merely 0.06. Malignant degeneration of an osteochondroma in the past, experiencing more pain, more depressive feelings and a higher BMI are negatively associated with patients’ PAL. Malignant degeneration of an osteochondroma has the second strongest association with patients’ PAL, reducing the BPAQ by 0.822. Having a paid job explains the highest unique variance (4.5%), followed by the experience of pain (2.9%) and depressive symptoms (2.2%).

**Table 3:** Summary of backward multiple linear regression analysis for physical activity level (BPAQ) as the dependent variable.

Independent variable	Unstandardized ß		ß	Adj. R^2^	F	95% CI	*p*	sr^2^	VIF
				0.221	16.989		< 0.001		
Work	0.843		0.234			0.5–1.2	< 0.001	0.045	1.176
Malignancy	− 0.822		− 0.133			− 1.4; − 0.2	0.006	0.018	1.025
BMI	− 0.040		− 0.119			− 0.07; − 0.007	0.017	0.013	1.095
NRS pain	− 0.126		− 0.183			− 0.2; − 0.05	< 0.001	0.029	1.221
HADS anxiety	0.060		0.134			0.003–0.115	0.036	0.010	1.831
HADS depression	− 0.105		− 0.206			− 0.2; − 0.04	0.002	0.022	1.976

Adj. R^2^ = explained variance, sr^2^ = squared semi-partial correlation, VIF = variance inflation factor.

*Adj. R*^*2*^ adjusted R^2^, *NRS* numeric rating scale, *HADS* Hospital Anxiety and Depression Scale.

### Associations with physical health-related quality of life

The final regression model for physical HRQOL is presented in Table 4. A total of 14 variables remained in the final model explaining 73.1% of the total variance. Higher anxiety levels and completion of secondary or tertiary education, compared to primary education, were associated with a better HRQOL, while all other factors were associated with a worse HRQOL. Pain-related disability explained the most unique variance (4.8%), followed by anxiety (1.8%) and age (1.3%).

**Table 4:** Summary of backward multiple linear regression analysis for physical component score (SF-36) as the dependent variable.

Independent variable	Unstandardized ß	ß	Adj. R^2^	F	95% CI	*p*	sr^2^	VIF
			0.731	66.697	53.8–64.4	< 0.001		
Educational level^a^								
Secondary	6.373	0.286			2.7–10.0	< 0.001	0.011	8.89
Tertiary	6.117	0.274			2.4–9.9	0.001	0.006	9.08
Surgery								
6–10	− 1.608	− 0.062			− 3.1; − 0.14	0.035	0.004	1.04
Pain locations^a^								
1–2	− 2.995	− 1.110			− 5.1; − 0.6	0.011	0.005	2.55
3–4	− 3.422	− 0.126			− 5.8; − 0.9	0.006	0.006	2.76
5–7	− 4.574	− 0.170			− 7.2; − 1.8	< 0.001	0.009	3.42
> 7	− 5.584	− 0.200			− 8.5; − 2.5	< 0.001	0.011	3.9
Age	− 0.084	− 0.122			− 0.12; − 0.04	< 0.001	0.013	1.21
BMI	− 0.232	− 0.110			− 0.4; − 0.1	< 0.001	0.010	1.24
NRS fatigue	− 0.674	− 0.157			− 1.0; − 0.3	< 0.001	0.011	1.94
NRS pain	− 0.760	− 0.178			− 1.2; − 0.4	< 0.001	0.012	2.58
PDI	− 0.295	− 0.399			− 0.4; − 0.2	< 0.001	0.048	3.32
HADS anxiety	0.447	0.162			0.3–0.6	< 0.001	0.018	1.45
FABQ	− 0.071	− 0.134			− 0.1; − 0.03	< 0.001	0.009	1.98

Adj. R^2^ = explained variance, sr^2^ = squared semi-partial correlation, VIF = variance inflation factor, ß = standardized regression coefficient.

*Adj. R*^*2*^ adjusted R^2^, *NRS* numeric rating scale, *PDI* pain disability index, *HADS* Hospital Anxiety and Depression Scale, *FABQ* Fear-Avoidance Beliefs Questionnaire.

^a^Dummy coded variables.

### Associations with mental health-related quality of life

Table 5 presents the final regression model of mental HRQOL. Eight factors remained in the model, explaining 61.8% of the total variance. Having a paid job, older age and greater pain-related disability were associated with a slightly better mental HRQOL. Higher levels of depression, anxiety and fatigue (NRS) were associated with a worse HRQOL. Patients who completed secondary or tertiary education had a lower mental HRQOL by at least 4.9 points compared to those who completed only primary education. Anxiety explained the most unique variance (9.4%), followed by depression (4.6%) and fatigue (2.5%).

**Table 5:** Summary of backward multiple linear regression analysis for mental component score (SF-36) as the dependent variable.

Independent variable	Unstandardized ß	ß	Adj. R^2^	F	95% CI	*p*	sr^2^	VIF
			0.618	69.656	56.9–66.9	< 0.001		
Age	0.73	0.113			0.02–0.1	0.002	0.011	1.13
Work	1.876	0.086			0.2–3.6	0.030	0.005	1.32
Educational level^a^								
Secondary	− 4.905	− 2.32			− 9.1 − 0.7	0.021	0.006	8.88
Tertiary	− 5.019	− 2.36			− 9.3 − 0.8	0.020	0.006	8.99
NRS fatigue	− 0.881	− 2.14			− 1.2; − 0.5	< 0.001	0.025	1.82
PDI	0.095	0.136			0.03–0.2	0.003	0.010	1.84
HADS anxiety	− 1.174	− 0.440			− 1.4; − 0.9	< 0.001	0.094	2.04
HADS depression	− 0.951	− 0.312			− 1.2; − 0.7	< 0.001	0.046	2.12

Adj. R^2^ = explained variance, sr^2^ = squared semi-partial correlation, VIF = variance inflation factor, ß = standardized regression coefficient.

*Adj. R*^*2*^ adjusted R^2^, *NRS* numeric rating scale, *PDI* pain disability index, *HADS* Hospital Anxiety and Depression Scale.

^a^Dummy coded variables.

## Discussion

To our knowledge, this is the first large-scale study on PAL and HRQOL of patients with MO that also provides insight into associated sociodemographic, illness-related and psychological factors. Our findings suggest that patients with MO experience significantly lower PAL and reduced physical HRQOL—compared to gender-matched healthy controls—while reporting similar mental HRQOL. An association between PAL and HRQOL could not be confirmed in our study population. However, several patient characteristics and psychological factors were associated with PAL and HRQOL in this study—many of which are clinically modifiable and may serve as targets for future interventions. Below, we discuss these findings in relation to our five pre-specified hypotheses.

Hypothesis (1): patients with MO have a lower HRQOL and PAL than healthy controls.

Patients with MO have a significantly lower PAL and physical HRQOL than gender-matched healthy subjects, but similar mental HRQOL. A minimal clinical important difference (MCID) of 2.5–5 points for physical HRQOL of patients with rheumatoid arthritis has been reported^44^, meaning that the lower physical HRQOL (> 5 points) for both men and women with MO compared to healthy subjects can be interpreted as a clinically worse state. To the best of our knowledge, the MCID of the BPAQ has not yet been reported in patients with musculoskeletal complaints, underscoring the need for future research.

Hypothesis (2): a higher PAL, after controlling for other factors, is significantly associated with a higher HRQOL in patients with MO.

A positive relationship between the PAL and HRQOL in MO could not be confirmed. While this contrasts with some prior findings in chronic musculoskeletal populations^25^, it is plausible that other clinical or psychological factors mediate or moderate this relationship in MO. One likely explanation is the heterogeneity of disease severity and functional limitations; in those with severe skeletal deformities and restricted joint motion, increased physical activity may not translate into functional gains or improved HRQOL. Further studies are needed to better understand the impact of disease severity in MO on the association between PAL and HRQOL.

Hypothesis (3): a higher BMI, higher pain and fatigue, and presence of psychological factors are negatively associated with the PAL.

The negative relationship between PAL and higher BMI, pain intensity, and depressive symptoms in MO-patients has been confirmed and is in accordance with other populations^45,46^. A longitudinal study on the reciprocal relationship between physical activity and depression showed that performing moderate to vigorous physical activity at least once a week is associated with lower depressed mood^45^. This should be targeted in the treatment plans of MO-patients. Patients’ PAL is positively associated with having a paid job and these patients had, besides a higher work-index, also a significantly higher PAL sport-, and leisure-index (*p* < 0.001) than patients who did not have a paid job. Being able to work seems an important contributor to patients’ PAL. More anxiety was also related to a higher PAL, while in contrast previous studies reported that lower levels of anxiety were related to higher levels of physical activity^46,47^. However, anxiety only contributes 1% of the total explained variance, which is rather negligible, and only 11.7% of the total patient sample has scores above the cut-off score (> 7) for anxiety^37^. Patients who experienced malignant degeneration of an osteochondroma into a chondrosarcoma in the past had a significantly lower PAL. These results are in line with findings on activity limitations after bone cancer^48^.

Hypothesis (4): female gender, higher BMI, comorbidity, higher pain and fatigue, physical disability and presence of psychological factors are negatively associated with physical HRQOL.

Our study confirmed this hypothesis for all factors except female gender. Ambiguity exists on whether MO affects men and women differently^3,8,9^. We hypothesized that female gender would be negatively associated with PAL and physical and mental HRQOL, but gender was not retained in any of the regression models. This suggests that, when controlling for other potential factors, gender is not an important contributor. In our study, significantly more comorbidities, neuropathic pain, higher level of pain, pain-related disability, fatigue, anxiety and lower physical HRQOL were reported by females. Of note, deformities and functional limitations, such as restricted joint motion, were not assessed in this study and cannot be compared between genders. These differences in phenotypes could be a confounding factor worth investigating in future research.

Previous studies in chronic musculoskeletal disease have established an association between activity limitations and a lower HRQOL^11,18^. In our study, greater pain-related disability was linked to reduced physical HRQOL in patients with MO. Although we did not find a direct relationship between physical activity level (PAL) and HRQOL, previous literature suggests that PAL may indirectly influence HRQOL through its effects on pain, fatigue, and psychological distress^49,50^. These findings imply that interventions aimed at reducing pain-related disability could potentially improve physical HRQOL in this patient group. Further research is needed to confirm these pathways. Consistent with other chronic musculoskeletal pain populations, fatigue intensity, age, BMI, and pain characteristics, such as more pain locations and pain intensity were also negatively related to patients’ physical HRQOL^10,50^. In contrast to the study of D’Ambrosi et al. (2017), more surgical interventions (6–10 interventions) were negatively related to physical HRQOL. Conversely, the lower HRQOL observed may indicate a more severe phenotype that might require additional surgical interventions, potentially explaining this outcome.

Higher anxiety scores were positively related to physical HRQOL, although only explaining 1.8%, while the opposite direction was expected^15^. The SF-36 PCS score increases with 0.447 for every point higher on the anxiety subscale, but the reason for this inversed directionality is unclear. Surprisingly, depressed mood and pain catastrophizing did not remain in the model^15,18^, but fear-avoidance beliefs were negatively associated with physical HRQOL^18^. On average, it seems that psychological factors, which are often reported and negatively associated with physical HRQOL in other chronic pain populations^13,18^, are less present in (or recognized by) patients with MO. Our sample is merely a cross-section of the total population and included patients who do not necessarily have high care needs. It is plausible that differences exist in the presence of psychological factors and their impact on HRQOL between patients with different care needs. Borsbö et al.^15^ identified four subgroups based on depression, anxiety, catastrophizing, pain intensity and duration in chronic pain patients (spinal cord injury, whiplash and fibromyalgia). Two subgroups who scored high on psychological factors reported lower HRQOL and more disability than the two subgroups who scored (relatively) low on psychological variables^15^. Subgrouping of patients based on psychological variables could have added value when investigating HRQOL.

The educational level was also positively related to physical HRQOL, in accordance with previous results of patients with chronic musculoskeletal pain^10,49,50^. Salaffi et al.^10^ hypothesized that a higher education may lead to more self-efficacy and consequently to a better self-management of disease-related symptoms and disability.

Hypothesis (5): female gender, being single, a lower educational level, malignancy, more surgical interventions, higher intensity level of pain and fatigue, and presence of psychological factors are negatively associated with mental HRQOL.

In the final mode female gender, being single, malignancy and amount of surgery were not retained. Conversely, higher levels of anxiety and depressed mood were related to lower mental HRQOL, similar to healthy subjects^15,51^. While pain intensity did not remain in the final model, fatigue intensity was negatively associated with mental HRQOL. This result shows that in MO like other musculoskeletal disorders, fatigue has a stronger impact than pain on mental HRQOL^16,52^. The negative association of a higher educational level with mental HRQOL is unexpected, as it seems to be positively related to self-efficacy^10,50^ and adequate coping skills which mediate the relationship between sense of coherence and mental HRQOL^53^. An underrepresentation of patients with a primary educational level (n = 2) and, surprisingly, slightly higher mental HRQOL experienced by these patients than those with a higher educational level in our sample seems to underlie this result.

In our sample, more pain-related disability was slightly positively related to mental HRQOL, while other studies in chronic pain patients found more perceived disability to be related to lower overall HRQOL^15,54^. A possible explanation could be that patients with MO have adapted to their situation^55^, keeping in mind that nearly all patients are diagnosed at a young age, and by the age of 12 years^7^ at the latest. Patients’ adaptation to their chronic illness and disability, and its relationship with HRQOL should be explored further. On average, the PDI score is relatively low. This may be partly explained by our recruitment from both an expertise center as through the patient association. It is possible that our patient sample had a lower intensity of care compared to patients who are hospitalized or had recent surgery.

### Limitations

First, reference scores from the general Dutch population used to compare patients’ PAL and HRQOL scores date from 1982^28^ and 1998^29^, respectively, and may be less representative of the current population.

Second, as this is a cross-sectional study, conclusions on causality cannot be drawn but require a longitudinal study.

Due to the recruitment procedure (expertise center and patient association) there is a possible under- or overrepresentation of persons who are asymptomatic or experience only mild symptoms. However, the number of completed questionnaires was high and patient characteristics showed a large degree of variability between patients, suggesting that both patients with no or mild and patients with severe symptoms were represented in our sample. Nevertheless, a potential selection bias cannot be excluded.

Even though the range of scores on psychosocial and symptom-related variables was large, we did not analyze our data based on known subgroups identified in other chronic disorders^15^. Due to the knowledge gap on aforementioned associations in patients with MO, a first exploration of the PAL and HRQOL, and associated patient-specific factors, symptom severity and psychological factors was necessary. A subsequent exploration of subgroups in the MO population could further clarify the association between psychological and symptom-related variables and the dependent variables (PAL and HRQOL). These additional insights could provide supplementary information for health professionals and support the development of individualized treatment programs.

Due to our study design, we relied on self-reported measures, which are susceptible to known biases such as recall bias. Subjective estimates of PAL may also differ from objectively measured data. For this study, we preferred a large sample size over the ‘objectivity’ of outcome measures. To minimize participant burden and maximize recruitment, we chose self-reported methods rather than multiple in-person assessments or additional objective measures.

Regarding our sample size, we did not perform a formal a priori power calculation (as noted in the Methods). Instead, we aimed for a convenience sample, as large as feasible within the study project’s constraints, to obtain the best possible estimates of our outcomes. We successfully enrolled a substantial cohort of individuals with MO—despite the rarity of this condition—which allowed us to develop robust regression models based on our theoretical framework. While additional associations may emerge with an even larger sample or an expanded set of variables, it is noteworthy that, to our knowledge, this is the largest adult MO population to date characterizing physical activity levels and health-related quality of life.

### Clinical implications

Considering our results, the management of pain, depressive feelings and lifestyle to lower BMI, seem important components to enhance patients’ PAL. A higher PAL in turn can lead to less disease-related symptoms, psychological factors and lower BMI, creating a reciprocal relationship. Additionally, employment seems to contribute strongest to patients’ PAL and should be addressed in the treatment of working-age patients.

The relationship between the PAL and HRQOL seems rather indirect. We hypothesize that improvement of pain and fatigue, psychological factors and lifestyle leads to a higher PAL, less activity limitations and consequently higher mental and physical HRQOL.

Psychological variables such as depressive symptoms and catastrophizing were present in our sample and showed substantial variability, with depressive symptoms being negatively associated with both PAL and mental HRQOL It cannot be excluded that subgroups exist within the MO population, some of whom experience a larger psychological burden. Consequently, we recommend that psychological variables be routinely assessed and addressed during patient treatment.

Age and educational level were also related to mental and physical HRQOL. Even though these demographic factors are ‘non-modifiable’, they are important to consider during treatment. Focusing on self-efficacy, adequate coping skills and goal-oriented care could enhance patients’ ability to handle disease-related symptoms, help them to adapt to their chronic illness and thus improve their HRQOL.

## Conclusions

This study provides important insights into the physical activity level and health-related quality of life of patients with MO, highlighting the significant impact of sociodemographic, illness-related, and psychological factors. Compared to healthy controls, patients with MO report lower physical HRQOL and PAL, which are associated with pain intensity, depressive symptoms and higher BMI. These results underscore the need for targeted interventions focusing on pain management, psychological factors, and lifestyle changes to improve both PAL and HRQOL in MO patients.

## Electronic supplementary material

Below is the link to the electronic supplementary material.

Supplementary Material 1

Supplementary Material 2

Supplementary Material 3

## Data Availability

The datasets used and/or analysed during the current study are available from the corresponding author on reasonable request.

## Abbreviations

BMI: Body Mass Index
BPAQ: Baecke Physical Activity Questionnaire
CIS: Checklist individual strength
DN4: Douleur Neuropathique en 4 Questions (Neuropathic Pain Diagnostic Questionnaire)
FABQ: Fear-Avoidance Beliefs Questionnaire
HADS: Hospital Anxiety and Depression Scale
HME: Hereditary multiple exostoses
HRQOL: Health-related quality of life
ICF: International classification of functioning
MCID: Minimal clinical important difference
MD: Mean difference
MO: Multiple osteochondromas
MCS: Mental component score (SF-36)
NRS: Numeric rating scale
PAL: Physical activity level
PCS: Pain catastrophizing scale
PDI: Pain disability index
SF-36: Short form-36
SF-36 PCS: Short form-36 physical component summary
