# Supplementary material for: Physical activity level and health-related quality of life in adults with multiple osteochondromas: a Dutch cross-sectional study
Source: Sci Rep. 2025 May 30;15:18990. doi: 10.1038/s41598-025-02812-3 (PMC12125288; doi:10.1038/s41598-025-02812-3)
Supplement: Supplementary file 1 — Supplementary Material 1 [file 41598_2025_2812_MOESM1_ESM.docx]

**Appendix 1: Full description of measurement instruments**

### **DEPENDENT VARIABLES**

#### **Physical activity level**

The level of habitual physical activity was measured with the Baecke Physical Activity Questionnaire (BPAQ). It consists of 16 items in three subscales: work, sports and leisure time activities index. The total score ranges from 3 to 15 and a higher score indicates a higher level of physical activity^1-3^.

#### **Health-related Quality of life**

The Medical Outcomes Study Short-Form 36 (SF-36) assesses general health and HRQOL. It consists of 36 items and eight subscales: physical functioning, role limitations due to physical health, role limitation due to emotional problems, energy, emotional well-being, social functioning, bodily pain and general health. One item measures a person’s perceived health change over the last year^4,5^. Subscale scores range from 0 to 100 and a higher score indicates higher levels of well-being and lower bodily pain^4,6^. Physical component (PC) and mental component scores (MC) were calculated according to their specific instructions and appropriate population-specific (Dutch) normative data^4,5^.

**EXPLANATORY VARIABLES**

#### **Sociodemographic information**

Age, gender, height, weight, education level, marital status, employment status, age of first surgery/diagnosis, amount of surgeries, their family history related to MO was obtained.

#### **Pain**

*Pain severity:* An 11-point numeric rating scale (NRS) ranging from 0 to 10 measured patients’ average pain severity, with a higher score indicating higher severity^7^.

*Pain disability:* The Pain Disability Index (PDI) measures the interference of average pain complaints on functioning in seven areas: family/home responsibilities. recreation, social activity, occupation, sexual behavior, self-care, and life-support activity. Each area is scored from 0 (no interference) to 10 (total interference), thus the total PDI score ranges from 0 to 70^8-10^.

*Neuropathic pain:* The Douleur Neuropathic 4 questions (DN4) is a 10-item diagnostic questionnaire that measures neuropathic pain. It consists of two parts, an interview (7 items) assessing pain characteristics and symptoms of abnormal sensations, and a clinical examination (3 items) assessing signs associated with neurological involvement. In this study, only the interview was included. A score of 4 or higher on the interview indicates the presence of neuropathic pain^11^.

#### **Fatigue**

*Fatigue severity:* An 11-point numeric rating scale (NRS) ranging from 0 to 10 measured patients’ average fatigue severity, with a higher score indicating higher severity^12^.

*Individual Strength:* The Checklist Individual Strength (CIS) is a 20-item questionnaire that measures four different areas of fatigue: fatigue severity, concentration, motivation and activity. Each item is scored on a 7-point Likert scale and added up a total score ranging from 20 to 140^13,14^. A higher score indicates more fatigue.

#### **Psychological factors**

*Anxiety and depression:* The Hospital Anxiety and Depression Scale (HADS) is a 14-item questionnaire measuring anxiety and depression complaints, but does not include complaints related to physical disorders. The anxiety and depression subscale consist both of seven items. All items are scored on a 4-point Likert scale with subscale scores ranging from 0 to 21 and a higher score indicating a worse feeling of anxiety or depression^15,16^.

*Catastrophizing*: The Pain Catastrophizing Scale (PCS) measures patients’ catastrophizing thoughts and feelings in relation to pain. It consists of 13 items and three subscales: rumination, magnification and helplessness. All items are scored on a 5-point scale and a total score ranging from 0 to 52 is calculated with a higher score indicating more catastrophizing^17-20^.

*Fear-Avoidance Beliefs:* The Fear Avoidance Beliefs Questionnaire (FABQ) measures patients’ fear-avoidance beliefs in relation to physical and work-related activities. It is a 16-item questionnaire with a 7-point Likert scale for all items and a maximal total score of 96. A higher score indicates a more avoidance beliefs^17,21^.

### **REFERENCES**

1 Baecke, J. A., Burema, J. & Frijters, J. E. A short questionnaire for the measurement of habitual physical activity in epidemiological studies. *Am J Clin Nutr* **36**, 936-942, doi:10.1093/ajcn/36.5.936 (1982).

2 Philippaerts, R. M., Westerterp, K. R. & Lefevre, J. Doubly labelled water validation of three physical activity questionnaires. *Int J Sports Med* **20**, 284-289, doi:10.1055/s-2007-971132 (1999).

3 Terwee, C. B., Bouwmeester, W., van Elsland, S. L., de Vet, H. C. & Dekker, J. Instruments to assess physical activity in patients with osteoarthritis of the hip or knee: a systematic review of measurement properties. *Osteoarthritis Cartilage* **19**, 620-633, doi:10.1016/j.joca.2011.01.002 (2011).

4 Aaronson, N. K. *et al.* Translation, validation, and norming of the Dutch language version of the SF-36 Health Survey in community and chronic disease populations. *J Clin Epidemiol* **51**, 1055-1068, doi:10.1016/s0895-4356(98)00097-3 (1998).

5 Ware Jr, J. E., Snow, K., Kosinski, M., & Gandek, B. *The SF-36 Health Survey: Manual and Interpretation Guide*. (1993).

6 McHorney, C. A., Ware, J. E., Jr., Lu, J. F. & Sherbourne, C. D. The MOS 36-item Short-Form Health Survey (SF-36): III. Tests of data quality, scaling assumptions, and reliability across diverse patient groups. *Med Care* **32**, 40-66, doi:10.1097/00005650-199401000-00004 (1994).

7 Hawker, G. A., Mian, S., Kendzerska, T. & French, M. Measures of adult pain: Visual Analog Scale for Pain (VAS Pain), Numeric Rating Scale for Pain (NRS Pain), McGill Pain Questionnaire (MPQ), Short-Form McGill Pain Questionnaire (SF-MPQ), Chronic Pain Grade Scale (CPGS), Short Form-36 Bodily Pain Scale (SF-36 BPS), and Measure of Intermittent and Constant Osteoarthritis Pain (ICOAP). *Arthritis Care Res (Hoboken)* **63 Suppl 11**, S240-252, doi:10.1002/acr.20543 (2011).

8 Soer, R. *et al.* Extensive validation of the pain disability index in 3 groups of patients with musculoskeletal pain. *Spine (Phila Pa 1976)* **38**, E562-568, doi:10.1097/BRS.0b013e31828af21f (2013).

9 Tait, R. C., Chibnall, J. T. & Krause, S. The Pain Disability Index: psychometric properties. *Pain* **40**, 171-182, doi:10.1016/0304-3959(90)90068-O (1990).

10 Tait, R. C., Pollard, C. A., Margolis, R. B., Duckro, P. N. & Krause, S. J. The Pain Disability Index: psychometric and validity data. *Arch Phys Med Rehabil* **68**, 438-441 (1987).

11 Van Seventer, R. *et al.* Validation of the Dutch version of the DN4 diagnostic questionnaire for neuropathic pain. *Pain Pract* **13**, 390-398, doi:10.1111/papr.12006 (2013).

12 Elera-Fitzcarrald, C. *et al.* Measures of Fatigue in Patients With Rheumatic Diseases: A Critical Review. *Arthritis Care Res (Hoboken)* **72 Suppl 10**, 369-409, doi:10.1002/acr.24246 (2020).

13 Vercoulen, J. H. *et al.* Dimensional assessment of chronic fatigue syndrome. *J Psychosom Res* **38**, 383-392, doi:10.1016/0022-3999(94)90099-x (1994).

14 Worm-Smeitink, M. *et al.* The assessment of fatigue: Psychometric qualities and norms for the Checklist individual strength. *J Psychosom Res* **98**, 40-46, doi:10.1016/j.jpsychores.2017.05.007 (2017).

15 Bjelland, I., Dahl, A. A., Haug, T. T. & Neckelmann, D. The validity of the Hospital Anxiety and Depression Scale. An updated literature review. *J Psychosom Res* **52**, 69-77, doi:10.1016/s0022-3999(01)00296-3 (2002).

16 Zigmond, A. S. & Snaith, R. P. The hospital anxiety and depression scale. *Acta Psychiatr Scand* **67**, 361-370, doi:10.1111/j.1600-0447.1983.tb09716.x (1983).

17 Crombez, G., Vlaeyen, J. W., Heuts, P. H. & Lysens, R. Pain-related fear is more disabling than pain itself: evidence on the role of pain-related fear in chronic back pain disability. *Pain* **80**, 329-339, doi:10.1016/s0304-3959(98)00229-2 (1999).

18 Osman, A. *et al.* Factor structure, reliability, and validity of the Pain Catastrophizing Scale. *J Behav Med* **20**, 589-605, doi:10.1023/a:1025570508954 (1997).

19 Sullivan, M. J. L. B., S. R.; & Pivik, J. The Pain Catastrophizing Scale: Development and validation. *Psychological Assessment* **7**, 524–532, doi:<https://doi.org/10.1037/1040-3590.7.4.524> (1995).

20 Van Damme, S. C., G.; Vlaeyen, J. W. S.; Goubert, L.; Van den Broeck, A.; Van Houdenhove, B. De Pain Catastrophizing Scale: Psychometrische karakteristieken en normering. *Gedragstherapie* **33**, 211-222 (2000).

21 Waddell, G., Newton, M., Henderson, I., Somerville, D. & Main, C. J. A Fear-Avoidance Beliefs Questionnaire (FABQ) and the role of fear-avoidance beliefs in chronic low back pain and disability. *Pain* **52**, 157-168, doi:10.1016/0304-3959(93)90127-B (1993).
