# Supplementary material for: Physical activity level and health-related quality of life in adults with multiple osteochondromas: a Dutch cross-sectional study
Source: Sci Rep. 2025 May 30;15:18990. doi: 10.1038/s41598-025-02812-3 (PMC12125288; doi:10.1038/s41598-025-02812-3)
Supplement: Supplementary file 2 — Supplementary Material 2 [file 41598_2025_2812_MOESM2_ESM.docx]

Checklist for Reporting Results of Internet E-Surveys (CHERRIES)

| **Item Category** | **Checklist Item** | **Explanation** | **Page Number** |
| --- | --- | --- | --- |
| **Design** | Target population | Dutch speaking adults aged 18 years and above diagnosed with Multiple Osteochondromas. | 6 |
|  | Sampling | Non-Probability Sampling  The sampling frame included patients identified through our hospital database as well as members of the national patient association. Because participants were specifically recruited from these two sources rather than through an unrestricted (“open”) invitation, the survey was not open. However, as no random sampling was employed and participation was voluntary, the final sample can be considered a convenience sample. | 6-7 |
| **IRB (Institutional Review Board) approval and informed consent** | IRB approval | The study was approved by the local Institutional Review Board of the OLVG (Reference No. WO 17.067). | 25 |
|  | Informed consent | An explanation of the survey with the statement of consent was presented in the beginning of the survey and only those who consented were allowed to continue the survey. | 6 |
|  | Data protection | The survey was administered using Castor, an Electronic Data Capture (EDC) platform certified for ISO 27001, ensuring compliance with recognized information security standards. Only the first author had access to participants’ email addresses, and all other identifying information was removed to produce a fully anonymized dataset. |  |
| **Development and pre-testing** | Development and testing | The survey was developed by the authors and an expert panel of rehabilitation physicians, informed by literature on quality of life, physical activity, pain, fatigue, and psychosocial factors in chronic diseases. It was pilot-tested among the authors and the chairman of the national patient association, then revised to improve clarity, validity, and reliability. |  |
| **Recruitment process and description**  **of the sample having access**  **to the questionnaire** | Open survey versus closed survey | The survey was a closed survey only accessible to those who got the survey link through their emails. |  |
|  | Contact mode | Participants were initially contacted at outpatient clinic visits or via the national patient association. After informed consent, a survey link was emailed to each participant. | 6-7 |
|  | Advertising the survey | The survey was advertised on the national MO patient association’s website. Interested individuals could contact the first author (IA) by phone or email. |  |
| **Survey administration** | Web/E-mail | The survey was hosted on Castor EDC; only link holders could access it, and responses were automatically recorded online. |  |
|  | Context | Castor EDC is an online survey platform available to all researchers over the world. It is HIPAA and GDPR compliant. Only those with approved access link or login ID can access the survey. |  |
|  | Mandatory/voluntary | Participation in the survey was voluntary |  |
|  | Incentives | none |  |
|  | Time/Date | The survey was in the field between May 2018 and December 2019 | 6 |
|  | Randomization of items or questionnaires | The questions were organized in sections for logical flow and structure. There was no need for randomization. |  |
|  | Adaptive questioning | Adaptive questioning was used where relevant. |  |
|  | Number of items | One questionnaire was presented per page, with the number of items varying according to each validated instrument (e.g. Baecke, CIS). |  |
|  | Number of screens (pages) | The questionnaire was distributed over 15 pages. A progress bar ranging from 0 to 100% was provided at the top of the pages to help respondents see their progress with the survey completion. |  |
|  | Completeness check | Completeness check was done prior to the analysis. The survey had minimal risk of missing data because the digital software prevented patients from skipping a question. |  |
|  | Review step | A back button was provided for respondents to review answers if needed. |  |
| **Response rates** | Unique site visitor | Not applicable |  |
|  | View rate | Not applicable |  |
|  | Participation rate | Not applicable |  |
|  | Completion rate | 392 participants, 342 completed surveys  Completion rate: 342/392= 87% |  |
| **Preventing multiple entries from the same individual** | Cookies used | Cookies were not used |  |
|  | IP Check | IP address was not required from respondents. |  |
|  | Log file analysis | No other techniques to analyze the log file for identification of multiple entries were used. |  |
|  | Registration | The closed survey system prevented duplicate entries from the same user. Once the survey was complete the survey was locked. |  |
| **Analysis** | Handling of incomplete questionnaires | The survey system was set up such that only completed questionnaires were recorded. |  |
|  | Questionnaires submitted with an atypical time stamp | There was no timeframe used as cut-off point for filling the questionnaire. |  |
|  | Statistical correction | No statistical correction procedures or weightings were used. |  |
