# Supplementary material for: Physical activity level and health-related quality of life in adults with multiple osteochondromas: a Dutch cross-sectional study
Source: Sci Rep. 2025 May 30;15:18990. doi: 10.1038/s41598-025-02812-3 (PMC12125288; doi:10.1038/s41598-025-02812-3)
Supplement: Supplementary file 3 — Supplementary Material 3 [file 41598_2025_2812_MOESM3_ESM.docx]

|  | **BAECKE PAL** | **Physical HRQOL** | **Mental HRQOL** | **Age** | **BMI** | **Nr. of surgeries** | **Malignancy** | **Paid work** | **PDI** | **FABQ** | **HADS Anxiety** | **HADS Depression** | **Pain locations** | **NRS pain** | **NRS fatigue** |
| --- | --- | --- | --- | --- | --- | --- | --- | --- | --- | --- | --- | --- | --- | --- | --- |
| **BAECKE PAL** | 1 |  |  |  |  |  |  |  |  |  |  |  |  |  |  |
| **Physical HRQOL** | 0,404 | 1 |  |  |  |  |  |  |  |  |  |  |  |  |  |
| **Mental HRQOL** | 0,096 | 0,054 | 1 |  |  |  |  |  |  |  |  |  |  |  |  |
| **Age** | -0,143 | -0,203 | 0,151 | 1 |  |  |  |  |  |  |  |  |  |  |  |
| **BMI** | -0,219 | -0,337 | 0,048 | 0,322 | 1 |  |  |  |  |  |  |  |  |  |  |
| **Nr. of surgeries** | -0,096 | -0,277 | -0,069 | -0,097 | 0,105 | 1 |  |  |  |  |  |  |  |  |  |
| **Malignancy** | -0,173 | -0,115 | -0,023 | -0,002 | -0,058 | 0,092 | 1 |  |  |  |  |  |  |  |  |
| **Paid work** | 0,363 | 0,37 | 0,182 | -0,275 | -0,228 | -0,119 | -0,088 | 1 |  |  |  |  |  |  |  |
| **PDI** | -0,42 | -0,783 | -0,323 | 0,097 | 0,236 | 0,256 | 0,145 | -0,365 | 1 |  |  |  |  |  |  |
| **FABQ** | -0,275 | -0,624 | -0,297 | 0,076 | 0,232 | 0,245 | 0,065 | -0,452 | 0,667 | 1 |  |  |  |  |  |
| **HADS Anxiety** | -0,084 | -0,194 | -0,723 | -0,106 | -0,027 | 0,036 | 0,032 | -0,117 | 0,367 | 0,29 | 1 |  |  |  |  |
| **HADS Depression** | -0,272 | -0,346 | -0,653 | 0,004 | 0,127 | 0,058 | 0,062 | -0,28 | 0,507 | 0,402 | 0,653 | 1 |  |  |  |
| **Pain locations** | -0,205 | -0,613 | -0,275 | -0,079 | 0,138 | 0,257 | -0,038 | -0,268 | 0,618 | 0,48 | 0,313 | 0,317 | 1 |  |  |
| **NRS pain** | -0,303 | -0,686 | -0,281 | -0,02 | 0,13 | 0,274 | 0,106 | -0,239 | 0,73 | 0,517 | 0,317 | 0,365 | 0,654 | 1 |  |
| **NRS fatigue** | -0,223 | -0,547 | -0,527 | -0,019 | 0,129 | 0,13 | 0,087 | -0,205 | 0,582 | 0,452 | 0,521 | 0,47 | 0,509 | 0,515 | 1 |

**Appendix 3. Correlation heatmap of key variables.**
